# Feasibility of intraoperative neuromonitoring and cortical/subcortical mapping in patients with cerebral lesions of highly functional localizations—pathway to case adapted monitoring and mapping procedures

**DOI:** 10.3389/fonc.2023.1235212

**Published:** 2023-11-23

**Authors:** Franziska Staub-Bartelt, Marion Rapp, Michael Sabel

**Affiliations:** Department of Neurosurgery, University Hospital Duesseldorf, Duesseldorf, Germany

**Keywords:** intraoperative monitoring, brain mapping, brain tumor, eloquent location, supratentorial brain tumor

## Abstract

**Background:**

Intraoperative neuromonitoring (IONM) and mapping procedures *via* direct cortical stimulation (DCS) are required for resection of eloquently located cerebral lesions. In our neurooncological department, mapping and monitoring are used either combined or separately for surgery of functional lesions. The study aims to provide a practical insight into strengths and pitfalls of intraoperative neuromonitoring and mapping in supratentorial functionally located infiltrating lesions.

**Methods:**

IONM and mapping techniques performed in eloquent located brain tumors were analyzed with a focus on neurological outcome and resection results obtained *via* MRI. Additionally, the surgeons’ view on obligatory techniques was explored retrospectively immediately after surgery. To evaluate the impact of the described items, we correlated intraoperative techniques in various issues.

**Results:**

Majority of the 437 procedures were performed as awake surgery (53%). Monopolar stimulation was used in 348 procedures and correlated with a postoperative temporary neurological deficit. Bipolar stimulation was performed in 127 procedures, particularly on tumors in the left hemisphere for language mapping. Overall permanent deficit was seen in 2% of the patients; neither different mapping or monitoring modes nor stimulation intensity, localization, or histopathological findings correlated significantly with permanent deficits. Evaluation of post-OP MRI revealed total resection (TR) in 209 out of 417 cases. Marginal residual volume in cases where total resection was assumed but MRI failed to proof TR was found (0.4 ml). Surgeons’ post-OP evaluation of obligatory techniques matched in 73% with the techniques actually used.

**Conclusion:**

We report 437 surgical procedures on highly functional located brain lesions. Resection without permanent deficit was adequately achievable in 98% of the procedures. Chosen mapping or monitoring techniques mostly depended on localization and vascular conflicts but also in some procedures on availability of resources, which was emphasized by the post-OP surgeons’ evaluation. With the present study, we aimed to pave the way to á la carte choice of monitoring and or mapping techniques, reflecting the possibilities of even supratotal resection in eloquent brain tumor lesions and the herewith increased need for monitoring and limiting resources.

## Introduction

Surgical gross total resection represents the gold standard according to therapeutical approaches of infiltrating brain tumors. The aim of the surgical intervention is an achievement of complete removal of the tumor as seen on the MRI scan and described as gross total resection in the literature in order to extend survival of the patients as this is directly linked to an extended overall survival in high-grade glioma patients (1–3). For low-grade glioma, it is well known that a residual tumor volume and extent of resection can predict the progression-free survival and time to malignant transformation (4, 5) as well as overall survival. Surgical resection in brain metastasis has also been the object of partly critical investigation. For this brain tumor entity too, surgical resection was found to be a significant factor for longer survival and preservation of functional status in many studies comparing the surgical approach and radiation therapy or radiation therapy alone. Patients that underwent surgery before radiation, regardless of the type of radiation (whole-brain or stereotactic radiosurgery), showed a significant longer patient survival and higher Karnofsky Performance Scale Scores (6, 7).

Thus, surgical therapy represents a main step in therapeutical concepts for cerebral lesions of different etiologies.

The most common highly functional supratentorial cerebral lesions comprise areas of motor and speech function. An important feature of gliomas and to some extent metastasis is the infiltrating zone. Here, functional tissue is infiltrated by malignant cells. Surgical intervention in this zone would inherently result in neurological/neurocognitive deficits, a non-resection in potential earlier recurrence. Thus, a possible definition of “eloquent” localization could be the ability to correlate a defined neurological/neurocognitive deficit with the destruction of this tissue by progression of the tumor or resection.

Maximum but safe resection is the superior aim in brain tumor surgery. In order to achieve the aimed resection but also to preserve functional status, as well as to provide the patient with optimal conditions for adjuvant therapies, intraoperative neuromonitoring and mapping using direct cortical and subcortical stimulation techniques have been well-established procedures in resection of tumors relating to motor pathways or speech areas and fiber tracts (8–12). In addition to motor and speech function, neurocognitive integrity has more and more become a target for intraoperative testing over the last few years (13–15).

There are different methods used for intraoperative monitoring (IOM) of neurological function. Transcranial electric stimulation (TES) can be used for motor-evoked potentials (TES-MEP) for monitoring of motor pathway integrity. Additionally, somatosensory-evoked potential (SSEP) monitoring to watch over sensory function can be performed. MEP monitoring can also be performed *via* direct cortical stimulation (DCS) by placing a strip electrode (SE) on the precentral gyrus allowing a continuous control of neurological motor function in asleep patients. DCS is also carried out by the usage of handheld monopolar or bipolar stimulation probes (16, 17), which enables the surgeon to map the functionality of the tissue. Bipolar stimulation is commonly used for awake mapping procedures, whereas monopolar mapping is particularly used for motor mapping for tumors located near or around motor pathways even though speech mapping is also performed at least on a research basis *via* monopolar mapping stimulation (18, 19). In summary, to date there are several options for mapping and monitoring procedures available, from which the surgeon can choose for special indications.

Over the past two decades, we have implemented the mentioned technical methods as well as awake and asleep resection protocols at our department. Instead of choosing one method over the other, we preoperatively determine what we think is the most suitable technique for preservation of neurological function. For reevaluation of the decisions, the present study was conceived in which we analyzed clinical and intraoperative data of 437 procedures in 400 patients that underwent surgery of eloquent brain lesions during a period of 4 years and complemented these data with subjective evaluations of the decisions made preoperatively by the surgeons that had performed surgery. All patients underwent surgery using either a single or combination of different mentioned intraoperative neuromonitoring and mapping methods. Additionally, we combined asleep and awake procedures. With this study, we aim to provide a deeper insight into strengths and pitfalls of intraoperative neuromonitoring and mapping in supratentorial eloquent brain tumors, especially gliomas and the hereby used methods at our institution.

## Patients and methods

In this single-center analysis (screening period 01/2019–01/23), we performed evaluation of intraoperatively collected data of neuromonitoring and mapping procedures in patients undergoing surgery for eloquently located supratentorial brain lesions. We complemented these data with sociodemographic data, clinical findings of preoperative and postoperative neurological status up to 6 months postoperative, and MRI studies for residual volume evaluation and neuropathological diagnosis. The study was approved by the local ethical committee (Study Number 2022-2242). Reporting of this study was according to the strengthening of the reporting of observational studies in epidemiology (STROBE) guidelines for observational studies (**Supplementary Material**).

### Patients

Inclusion criteria for the present analysis were (1) surgical intervention in patients >18 years between January 2019 and January 2023. (2) Intraoperative monitoring and/or mapping devices were used. (3) The addressed lesion was located supratentorial. Procedures for posterior fossa or spine surgery were excluded. Availability of IONM or mapping data was also taken into consideration as to some extent some data were missing in some of the cases. However, missing of few data of individuals did not lead to exclusion. The number of procedures is given for all single analyses.

### Data collection

#### General data

Sociodemographic data, neuropathological results, and information on medical/surgical history, if applicable, were taken from the local patient administration system. Surgical history was divided into four categories: (1) primary surgery, (2) recurrent surgery with neuropathological confirmation of recurrent disease, (3) recurrent surgery without neuropathological confirmation of recurrent disease, (4) 2nd-look surgery.

Neuropathological results if obtained before introduction of WHO 5 Classifications of Central Nervous System Tumors 2021 (20) were adapted according to the new classification.

#### Neurological outcome

Patients underwent initial neurological examination at timepoint of admission; this was defined as timepoint “pre-operative”. Postsurgery patients underwent multiple examination, especially in case of any new neurological deficit. For the present analyses, we consistently used the examination at timepoint of dismission for definition of timepoint “postoperative”. Furthermore, patients with new neurological deficit in the postoperative state were followed up at around 3 months (“3-month FU”) and 6 months (6-month FU). Neurological examination was performed by different specially trained team members who also carried out awake procedure preparation and awake procedure testing intraoperatively. Our protocol includes a detailed questionnaire about general condition and health-related problems as well as a detailed neurological examination of cranial nerves, motoric and sensory testing, and, if applicable, speech testing as described further on.

#### Monitoring and mapping data

Monitoring and mapping data were obtained using the following technical devices with a described standard setup for different monitoring/mapping techniques.

*ISIS Xpert and C2 Xplore (inomed Medizintechnik GmbH, Emmendingen, Germany, NeuroExplorer Software Version 6)*.

monitoring
SSEP (ISIS only)
TES-MEP (ISIS only)
DCS MEP (ISIS only, four to six contact subdural strips).

mapping
Cortical and subcortical with monopolar probe
cortical and subcortical with bipolar probe

In cases where the C2 Xplore device was used, amperage of bipolar stimulation is given in the numbers of ISIS Xpert device as there are other technical nuances between those devices leading to different settings. For better comparison, we standardized the data obtained.

*Ojemann Cortical Stimulator (Integra LifeSciences)*

mapping
Cortical and subcortical with bipolar probe

#### Awake status

Additionally, surgical protocols were screened for stimulation details and information on awake status and time of awake condition if applicable. Awake status was divided into following subcategories: “awake,” “not adequately awake,” “not awake”. In cases where awake surgery was planned but not conducted due to non-compliance of patients or other reasons (“not adequately awake”), the procedure was categorized in awake surgery status for statistical analyses.

More detailed technical data such as monitoring/mapping devices and technical setups are reported in the **Supplementary Information**.

#### Choice of adequate mapping/monitoring or speech testing

At patients’ presentation and when indication for surgery is set due to radiological and or clinical findings, we take a deep look into MRI scans and decide as a team of the leading surgeon and assistant surgeon as well as monitoring staff which technique to use in this special case. There has to be careful consideration of clinical and technical examination results in order to choose the right methods for the particular cases. The day before surgery, team members of the neurooncological team will talk to the patient through the procedure and perform neurological examination of the patient with evaluation of cranial nerves, motor and sensibility deficits, and general symptoms as headaches and perform a quick screening of speech disturbances. If there are any conspicuousness about speech deficits in the screening, the whole testing battery that we defined at our department is useful for efficient speech testing is performed. Details about the speech testing are described below.

#### Language testing

When language testing was performed, different tests were conducted with some items taken from Aachen Aphasia Test (21). Baseline testing was performed the day before surgery in order to have comparable data for intraoperative testing. Furthermore, all patients underwent postoperative testing at least at one time point in the postoperative state until they were discharged.

The following dimensions of language skills were tested pre-intra- and postoperatively in order to evaluate patients’ speech affection:

##### Spontaneous speech

Patients are motivated to talk about a topic of their choice. This is done to test semantic aspects of the patients’ speech, articulation, phonology, and syntax in general.

##### Token test

Testing language comprehension by showing and matching geometrical shapes of different sizes and colors.

##### Free reading

By reading the written language comprehension is tested.

##### Picture naming

Analysis of the designation of images of colors, objects, or actions.

##### Pyramids and palm trees test

Test for semantic memory used to detect language impairment. The test uses iconic images to determine the degree to which a subject can access meaning from pictures and words.

#### Surgeons’ postoperative evaluation

In order to compare chosen methods with a postoperative reevaluation, we retrospectively performed inquiries of surgeons concerning assessment of obligatory monitoring/mapping in the present case. Obligatory modes were divided into the following 13 categories: monopolar stimulation, bipolar stimulation, combination monopolar/SSEP/MEP, combination monopolar SSEP/MEP/SE, combination monopolar/bipolar, combination monopolar/bipolar/SSEP/MEP, combination monopolar/bipolar/SSEP/MEP/SE, combination bipolar SSEP/MEP/SE, SSEP/MEP/SE only, monitoring/mapping (m/m) not needed, m/m not applicable/no resection, used methods inconclusive.

The chosen method for each specific case served as preoperative evaluation and was not documented separately.

Procedures were led by three senior surgeons with each more than 10–25 years of experience in the field of brain tumor surgery and intraoperative monitoring/mapping procedures. Senior surgeons were accompanied by residents with different experiences in brain tumor surgery.

#### Residual volume (MRI)

For evaluation of residual tumor volume, results of postoperative conducted MRIs were screened. All MRIs were carried out within 72 h postsurgery. We defined four groups for result description:

(1) intraoperatively defined macroscopic total resection and total resection in postoperative MRI, (2) intraoperatively defined macroscopic total resection and residual tumor volume in postoperative MRI, (3) intraoperatively defined macroscopic residual tumor volume and residual tumor volume in postoperative MRI, and (4) no MRI. Residual volume was either calculated by the reporting radiologist or if missing by the study team under usage of a volumetry tool in the local radiology information system (SECTRA Workstation 101, IDS7, Version 24.1, Sectra AB, Sweden, 2022). Results of residual tumor volume are stated in milliliters. Residual volumes less than 0.1 ml were defined as total resection.

#### Statistical analyses

All statistical analyses were conducted using IBM SPSS Statistics Version 26 (IBM Corporation, USA). Obtained results were statistically analyzed by using chi-square test for nominal variables. Group comparison was performed by univariate analyses of variance by (ANOVA), and *post-hoc* tests were adjusted using Bonferroni correction. Additionally, we carried out correlation calculation under usage of Pearson correlation. Statistical cutoff stated as p-value for all results was set at 0.05.

## Results

### General data

Overall, we included 437 surgical procedures in 400 patients (47% women, n = 188; 53% men, n = 212) over a period of 48 months in the present analyses. There were 27 patients who underwent surgery twice and five patients who had triple surgery during the observation period.

The mean age of patients at surgery was 56.6 years [± SD 14.9, range 20–90 years]. If patients underwent more than one surgical procedure, age at first recorded surgery was enclosed in the reported data.

68% of surgeries were primary cases (n = 296). One-third were recurrent surgeries with neuropathological confirmation of recurrent disease (n = 121). In 3% (n=12), recurrent surgery revealed no recurrent disease but showed other diagnoses, for example radio necrosis or reactive tissue changes. Eight (2%) procedures were labeled as second-look surgery in patients with significant residual tumor volume in postoperative MRIs due to different reasons.

One patient underwent primary surgery without mapping/monitoring and showed residual volume in the postoperative MRI; therefore, second-look surgery was advised. Four patients showed different impairments under subcortical stimulation (one patient anomia with 2 mA bipolar subcortical stimulation, one patient >70% deterioration in the picture naming test as well as under subcortical bipolar stimulation, one patient who underwent monopolar subcortical mapping with a 2-mA resection limit achieved, and one patient who showed a significant increase in SSEP latencies and therefore resection had to be stopped).

In two patients, primary surgery was finished under expectance of total resection with no link to functional limits. One patient underwent primary surgery under expectance of debulking as resection could only be achieved under speech monitoring, but speech testing preoperatively showed too much effect for reliable intraoperative testing. After recovery from primary surgery, awake surgery was evaluated as soon as possible; therefore, a second-look surgery with indented total resection was performed.

A total of 235 procedures were performed on lesions in the left hemisphere (54%), 196 were right-hemispheric tumors (45%), and 6 were located elsewhere (1%, rostrum, splenium, bifrontal).

Majority of neuropathological diagnoses were high-grade glioma (glioblastoma, IDH-wild type, MGMT methylation positive or negative) with 191 procedures (43.7%). IDH-mutant astrocytoma (WHO 1–4) and cerebral metastases were each diagnosed in 86 procedures (19.7%). Oligodendroglioma, IDH-mutant 1p/19q co-deletion (WHO 2 + 3), was diagnosed in 38 procedures (8.7%).

For a summary of cohorts’ complete general data results, please refer to **Table 1**.

**Table 1:** Summary of cohorts' general data.

AGE (y)	
Mean	56.6 [SD ± 14.9]
Range	20–90
	n = 400
SEX	
Female	188
Male	212
DIAGNOSIS	
Astrocytoma IDH-mutant (2-3)	80
Astrocytoma IDH-mutant (4)	6
Glioblastoma, IDH-wild type (4) MGMT −	105
Glioblastoma, IDH-wild type (4) MGMT +	86
Oligodendroglioma (2-3)	38
Diffuse hemispheric glioma	1
Cerebral metastasis	86
Aggressive NHL	7
Meningioma	1
Atypical meningioma	4
High-grade neuroepithelial tumor	1
Low-grade neuroepithelial tumor	3
Dysembrioplastic neuroepithelial tumor	1
Ganglioglioma	2
Radiation necrosis	2
Reactive tissue changes	10
Chronic inflammatory tissue changes	1
Florid inflammatory demyelinating	
CNS lesion	1
Cerebral toxoplasmosis	2
SURGICAL HISTORY	
Primary surgery	296
Recurrent surgery with diagnosis of	121
recurrent disease	
Recurrent surgery without diagnosis of	12
recurrent disease	
Second look	8
LOCALIZATION	
Left hemisphere	235
Right hemisphere	196
Other	6

### Awake status

**Figure 1:**
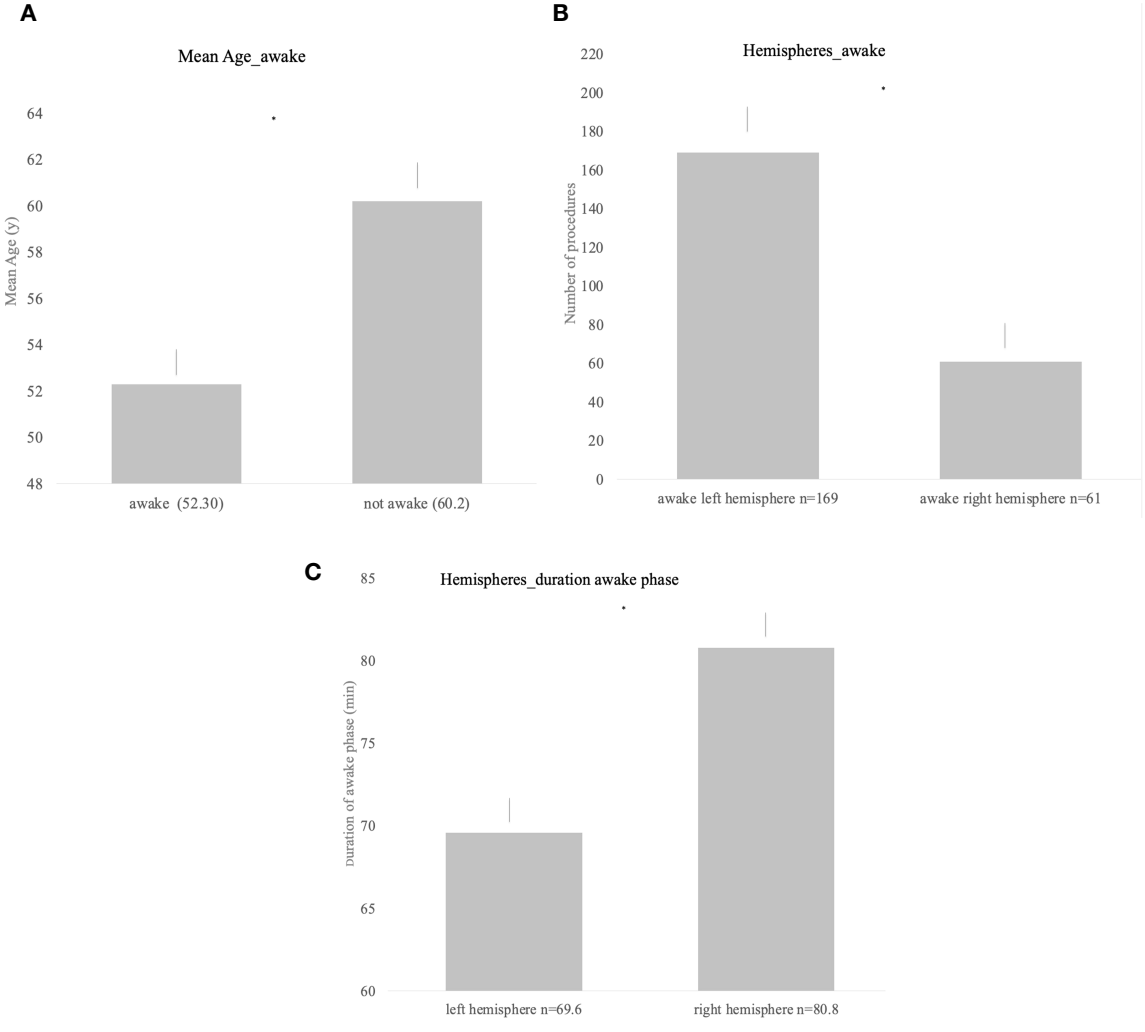
**(A)** Significant difference in the mean age and awake status (p < 0.001). **(B)** Awake status for procedures of both hemispheres; left hemispheric lesions were significantly more often operated under awake settings (p < 0.001). **(C)** Duration of the awake phase for both hemispheres showing significantly longer awake duration in right hemispheric lesions (p = 0.0023).

Overall, 53% (n = 233) of procedures were conducted as awake surgery or were at least planned as awake surgery. Out of 233 planned awake procedures, 36 were categorized as “not awake adequately” (15%). Most frequent localizations for awake surgery in left hemispheric tumors were frontal, temporal, fronto-temporal, and parietal lesions, in the right hemisphere most commonly right parietal tumors followed by temporal and frontal lesions. Patients that underwent planned awake surgery were significantly younger compared with the non-awake patients with a mean age of 52.3 years in the awake group vs. 60.2 years in the non-awake group (p < 0.001, **Figure 1A**).

Awake status and duration of awake status differed significantly according to the hemisphere. While lesions located in the left hemisphere were more frequently planned and conducted as awake procedure (p < 0.001, **Figure 1B**), the duration of the awake phase was significantly longer when performed on lesions in the right hemisphere (left = 69.6 min [± SD 25.2] vs. right = 80.8 min [± SD 27.1], p = 0.023, **Figure 1C**).

### Intraoperative monitoring/stimulation data

#### TES-MEP and SSEP monitoring

SSEP monitoring was conducted in 234 and TES-MEP in 260 procedures. The range of stimulation for the present cohort for left and right medianus SSEP monitoring was 0.8–20 mA and for tibilias SSEP on the right side 0.5–30 mA and for the left side 1.7–30 mA. For MEP monitoring, maximum stimulation of 220 mA at a band-pass filter between 250 and 500 Hz or a maximum of 100 mA at 500 Hz was used. For upper extremities, the range of stimulation was 40 to 80 mA and for lower extremities 60–110 mA in the present cohort.

**Figure 2:**
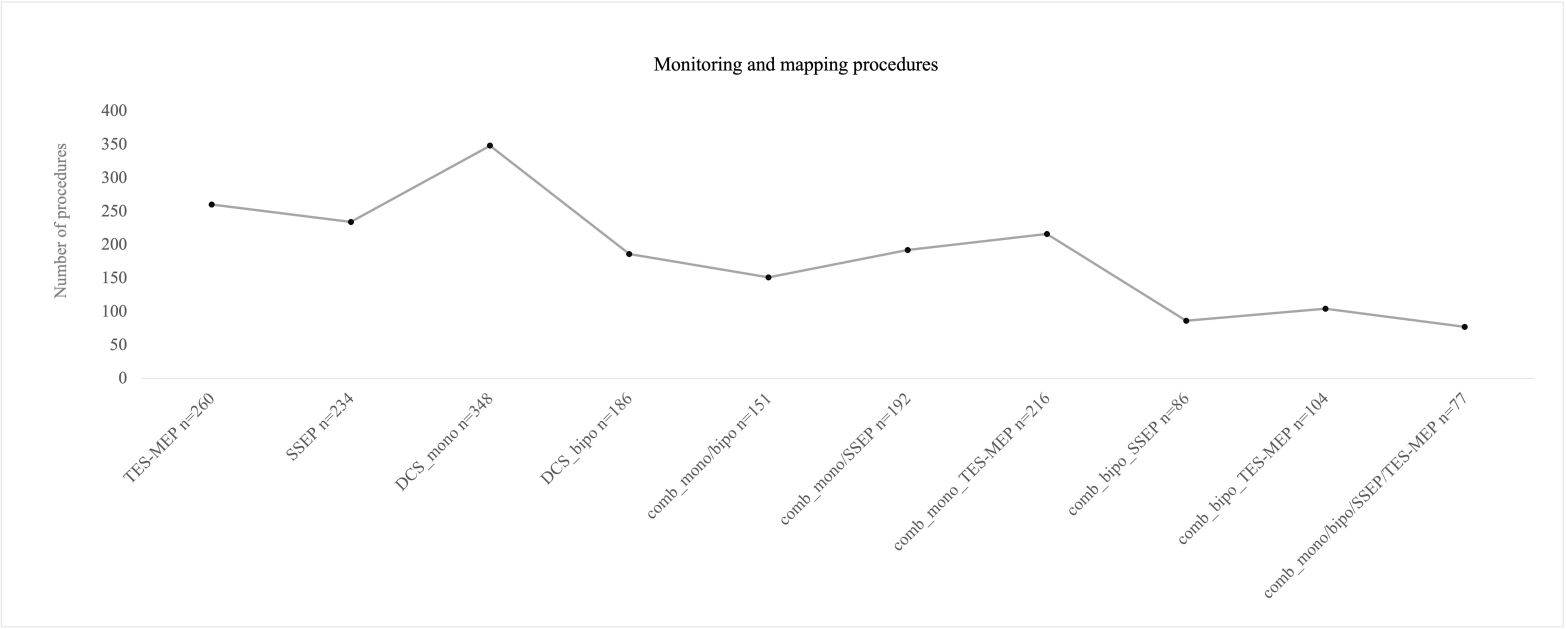
Number of procedures for different monitoring/mapping modes that were used intraoperatively. Mostly monopolar stimulation was used. The combination (“comb”) of monopolar and bipolar mapping plus monitoring was the least combination that was used intraoperatively.

**Figure 3:**
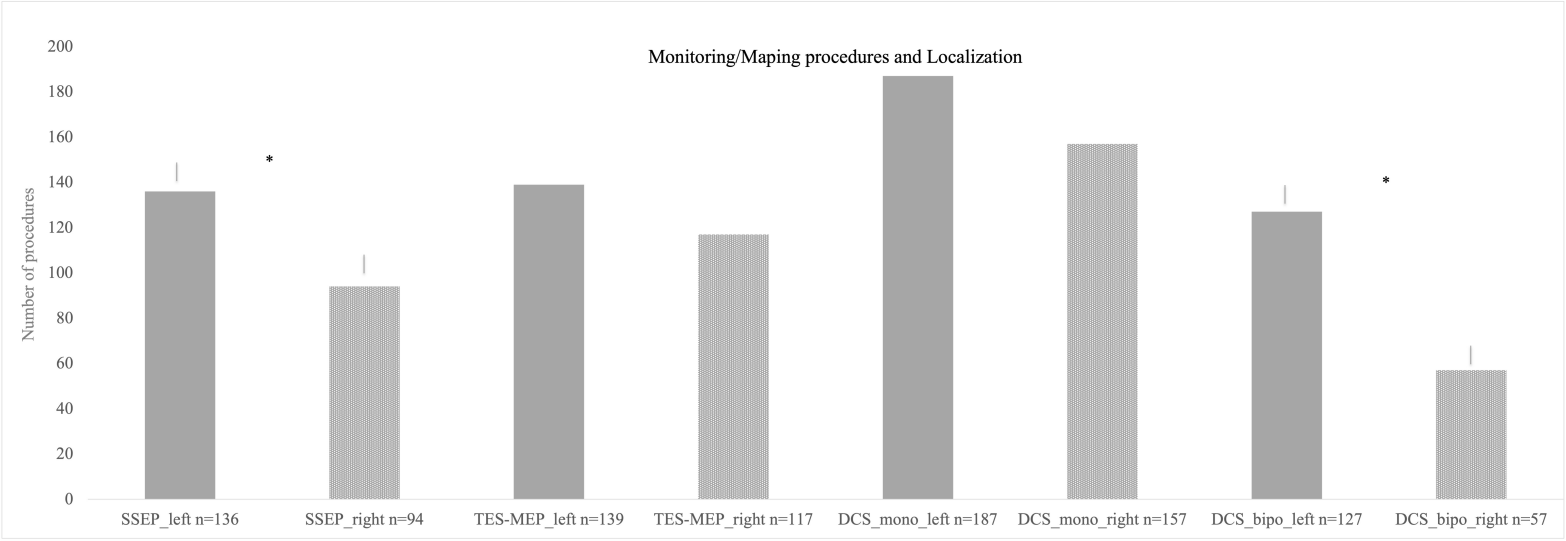
Different monitoring and mapping procedures separated according to localization. Monopolar stimulation was mostly used on left hemispheric lesions followed by monopolar stimulation on right hemispheric procedures. The least used method was bipolar stimulation for right hemispheric lesions. * = significant results.

**Figure 4:**
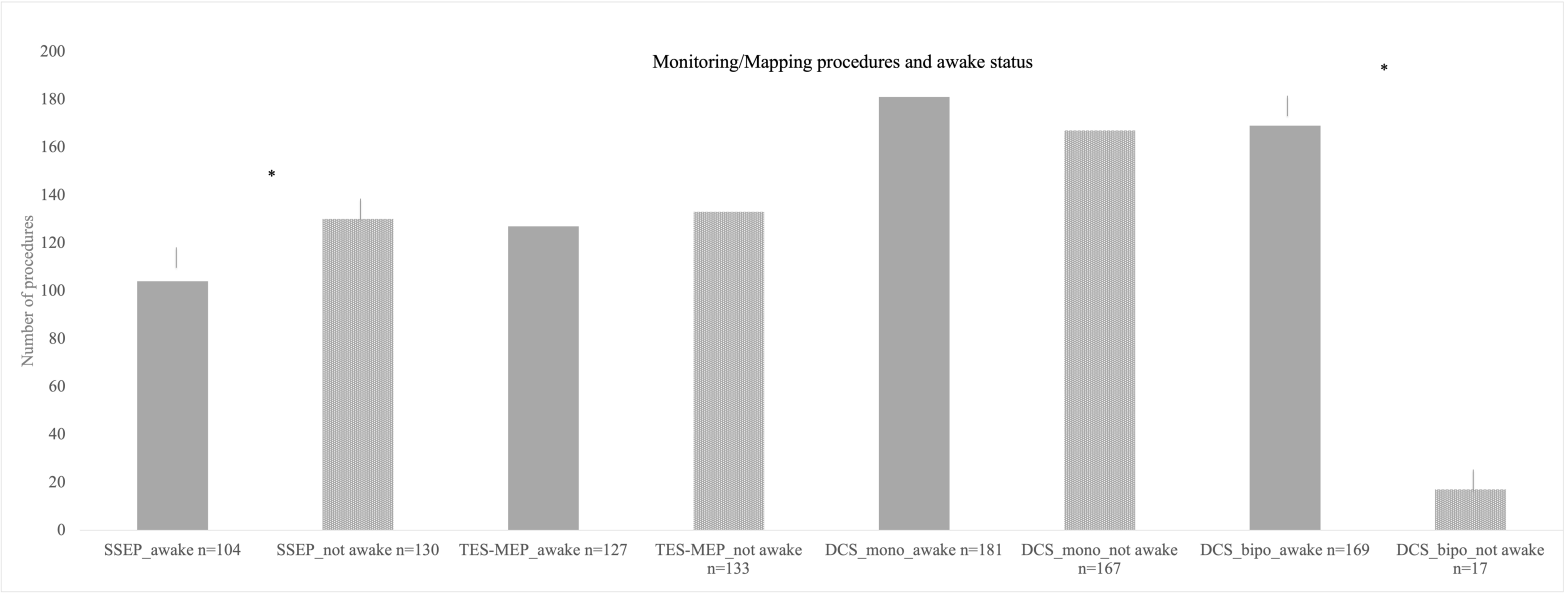
Different monitoring and mapping procedures here parted in accordance by awake status. Here, also monopolar mapping was most commonly used either awake or asleep with no significant difference in number of procedures. Bipolar stimulation in the asleep status was used least in the cohort. * = significant results.

SSEP and MEP monitoring *via* SE was conducted in 172 cases (**Figure 2**). SSEP monitoring was significantly more frequently used in left hemispheric lesions (n = 136 vs. 94, p = 0.007), whereas usage of TES-MEP did not significantly differ concerning localization (**Figure 3**). SSEP monitoring was significantly more often used in asleep surgery status (p < 0.001 SSEP, **Figure 4**).

Furthermore, we analyzed EEG documentation for intraoperative seizures; EEG data were available for 260 patients. Seizures were observed in 22 patients either *via* EEG only or by EEG and clinical determination (8%). There were 13 patients with seizure occurrence (59%) who underwent an awake procedure. Preoperative seizure did not increase the risk for intraoperative seizures (p = 0.854), but bipolar stimulation significantly correlated with occurrence of intraoperative seizures (p = 0.008) whereas monopolar stimulation did not.

#### Monopolar/bipolar mapping

Monopolar mapping was conducted in 348 procedures (**Figure 2**). Stimulation ranged from 0.5 to 20 mA. Epidural stimulation in 127 cases (36%) was conducted with a mean current of 10.6 mA [± SD 4.2]. In the vast majority of surgeries, a cortical stimulation was performed with 329 cases (96%) and a mean current of 7.6 mA [± SD 4.4]; subcortical stimulation was found in 302 surgeries (87%) with a mean current of 3.4 mA [± SD 3.2], ranging from 0.2 to 20 mA.

The proportion of awake procedures in monopolar mapping procedures was 52% with 181 cases (**Figure 4**).

There were 186 procedures conducted under usage of bipolar stimulation (**Figure 2**). Bipolar cortical mapping was found in 91% (n = 169) with a mean current of 2.2 mA [± SD 1.4, range 1–3 mA] and subcortical stimulation in 73% (n = 136) with a mean current of 2.1 mA [± SD 1.0, range 0.8–3 mA]. In the awake setting, 169 patients underwent bipolar stimulation (91%, **Figure 4**). If clinical evaluation, in case of awake surgery, or MEP/SSEP monitoring allowed so, resection was stopped at a minimum of 1 mA when performing subcortical mapping.

In 151 cases, both monopolar and bipolar stimulation were performed, thereof a total of 91% (n = 136) proceeded in an awake setting (**Figure 2**).

During both mapping procedures, awake surgery status was more frequently planned than asleep procedures (monopolar stimulation p = 0.026, bipolar stimulation p ≤ 0.001, **Figure 4**).

Localization did not correlate significantly with use of monopolar mapping; however, bipolar mapping was used more frequently for left hemispheric tumors (p ≤ 0.001, **Figure 3**).

### Surgeons’ evaluation of obligatory stimulation mode

Most frequently monopolar mapping (n= 172), bipolar mapping (n= 98), and combination of monopolar/bipolar mapping (n= 85) were designated as obligatory stimulation modes. In only 10 procedures, combination of all mapping and monitoring techniques was seen as obligatory (four lesions left frontal, temporal and fronto-temporal, six lesions right fronto-parietal, parietal and fronto-temporal); however, SSEP/MEP monitoring was seen obligatory in around 16% of all procedures, independently from mapping procedures that were performed, but most commonly in right hemispheric lesions with monopolar stimulation combined with SSEP/MEP monitoring.

**Figure 5:**
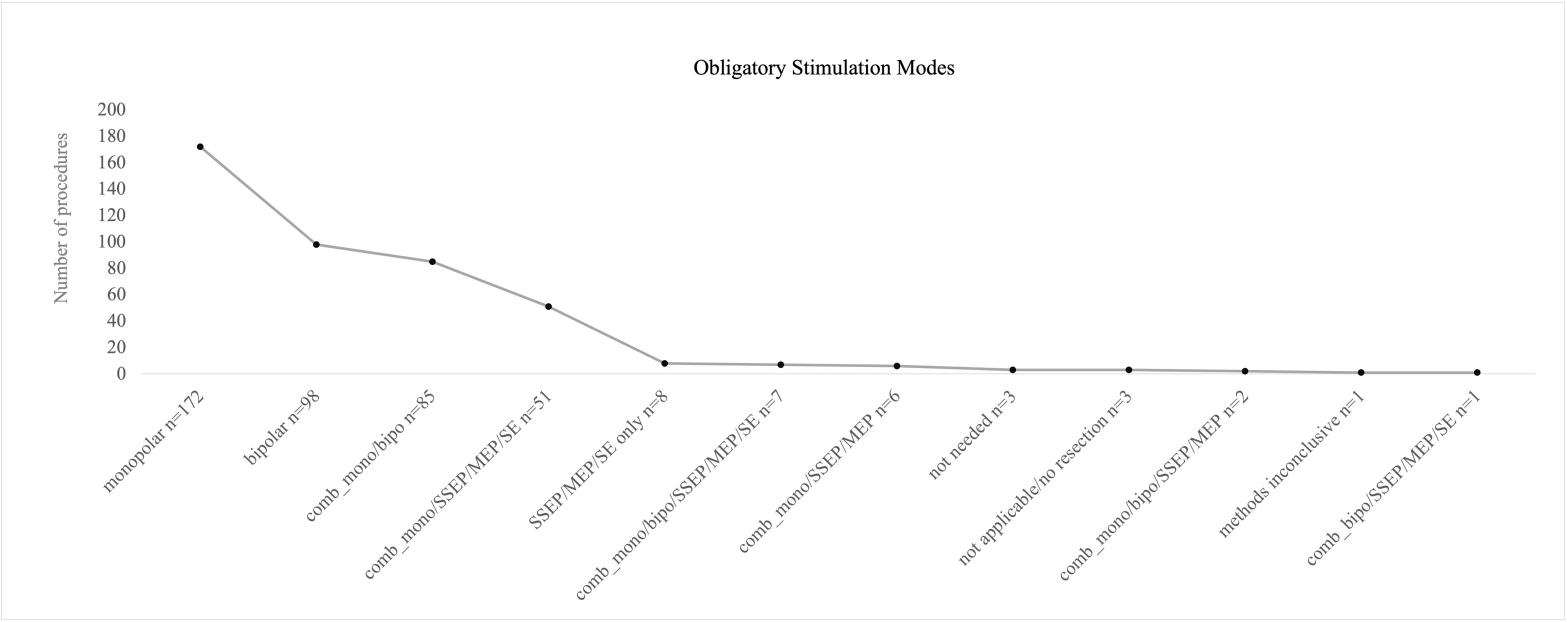
Postoperative evaluation of obligatory mapping or monitoring modes by the operating surgeon. Monopolar, bipolar, and a combination of both mapping procedures were mostly stated as “obligatory” for the preceding surgery. Full technical equipment with a combination of mapping and monitoring was less frequently recalled as obligatory.

In eight cases, SSEP/MEP monitoring only was evaluated as required without any mapping procedure. Three procedures were performed without any requirement of monitoring or mapping due to intraoperative non-functional localization (0.7%). Two surgeries were terminated without any intervention. In one procedure, all methods used were rated as inconclusive with no benefit for safe resection (**Figure 5**).

A significant correlation between localization of tumor and postoperative stated obligatory stimulation modus was found (p = < 0.001).

Monopolar obligatory mapping was found to be essential in right hemispheric tumors more often than left hemispheric ones; on the contrary, bipolar mapping and combination of monopolar/bipolar mapping were evaluated as essential more often on left hemispheric lesions.

Incongruency of the obligatory method according to surgeons’ evaluation and the intraoperatively used method was more often seen with obligatory bipolar mapping (23% bipolar mapping, 10% for monopolar mapping).

### Clinical outcome

In 279 cases, preoperative deficits were noticed (64%), mostly motor deficits (n = 70), speech disorders (n = 58), affection of vision and or cranial nerves (n = 28), and behavioral changes (n = 28). Additionally, preoperative seizures were observed in 49 cases (11%).

**Figure 6:**
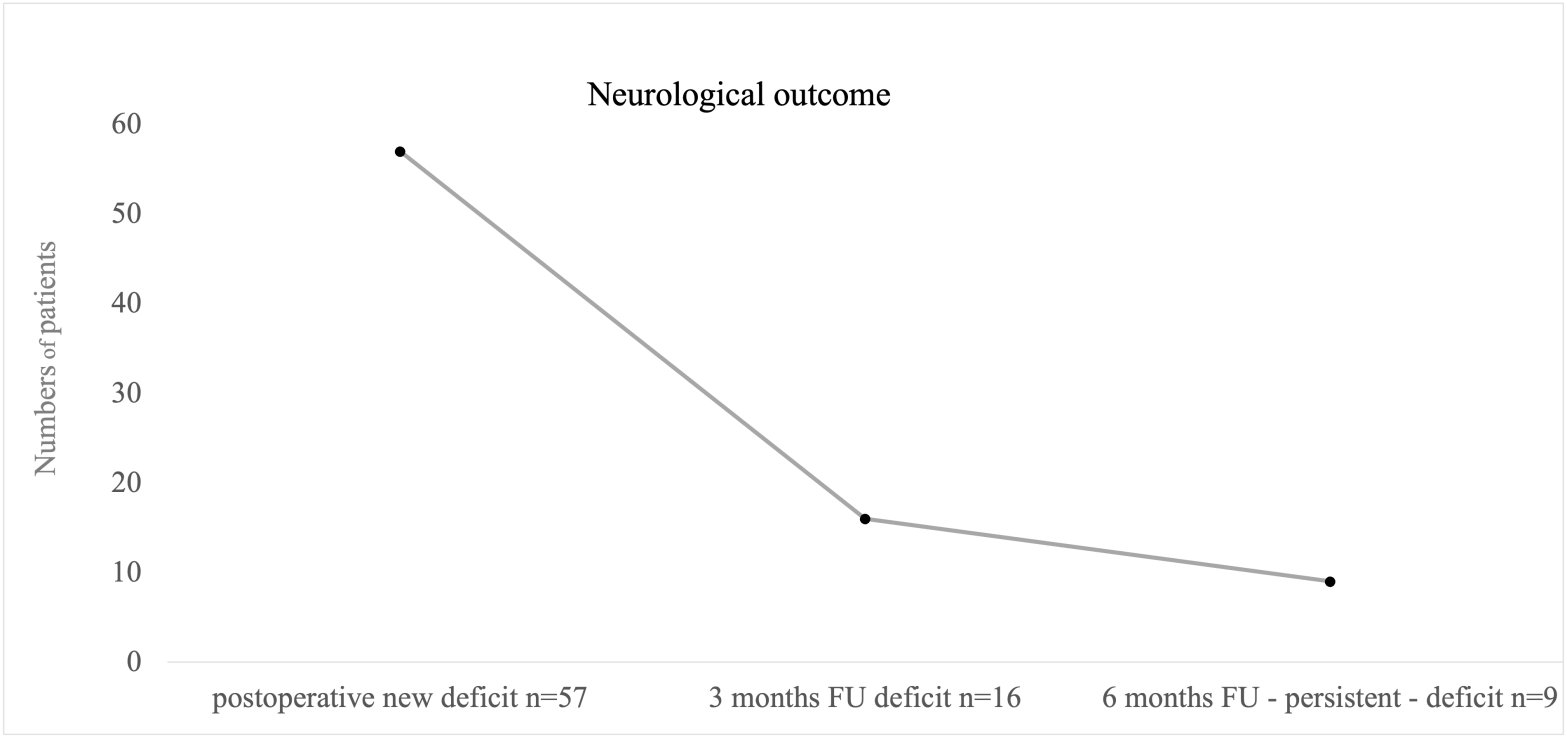
Illustrating cases with postoperative and persistent new neurological deficits. We defined “persistent deficit” as neurological impairment after 6 months postoperative. Nine patients suffered from persistent deficits after surgical intervention; mostly permanent speech disorders were seen.

A new postoperative neurological impairment was seen in 57 patients (13%, **Figure 6**); seven patients died shortly after resection. However, deaths were not directly related to surgical complications. Majority of neurological deficits (61%) were seen after procedures on left hemispheric tumors, and right hemispheric surgeries led to postoperative new deficits in 35% of the cases. 4% occurred in other locations.

A total of 24 patients suffered from high-grade hemiparesis, one patient showed sensory deficits as new and leading symptoms, 22 patients presented a new speech disorder affecting either sensory or motoric aspects of speech, and global aphasia as the combination of both was seen in one patient. Three patients presented new impairment in facial motoric or vision. Six patients had a combined deficit of speech and motor function.

At first follow-up after 3 months postoperative, persistent neurological deficits in 16 cases out of 56 were reported (4%, n = 411, **Figure 6**). At 6 months of follow-up, neurological deficits were still seen after nine procedures, an overall of 2% concerning all procedures, and were defined as permanent deficit by that time (n= 406, **Figure 6**) with five patients with persistent motor impairment and four speech disturbances. Overall, 13 patients had died within 6 months postsurgery (2%).

Permanent deficits occurred independently from diagnosis (p = 0.958) or localization (p = 0.271). Significantly increased risk of death was seen in cases with preoperative neurological deficit (p = 0.030). Surgical status “awake” significantly correlated with direct postoperative speech deficits not motor deficits (p = 0.003). Overall evaluation of persistent deficit after 6 months however revealed no significant influence by awake or asleep status (p = 0.593).

Correlation of stimulation/monitoring modes and postoperative neurological deficit revealed no significant results for SSEP monitoring (p = 0.341), TES-MEP (p = 0.659), and bipolar stimulation (p = 0.061), but monopolar stimulation with p = 0.007. Permanent deficit at 6 months did also only significantly correlate with usage of monopolar stimulation (p = 0.012). At last, neurological impairment and obligatory stimulation mode turned out to be not significantly related (p = 0.109).

We examined postoperative MRI scans of patients who experienced new neurological impairments after surgery. In our analysis, we identified indications of infarction or postoperative bleeding in 21 MRI scans (comprising 17 cases of infarction and 4 cases of bleeding). Notably, most infarctions were very and relatively small (n = 15) and are not assumed to be a potential reason for neurological impairment. In two patients, the infarction may have contributed to their postoperative and later permanent deficits. In a specific case, a patient exhibited a basal ganglia infarction and subsequently experienced postoperative motor deficits. Additionally, in the second patient, a relatively extensive territorial infarction occurred, leading to motor deficits as well.

### Resection results

**Figure 7:**
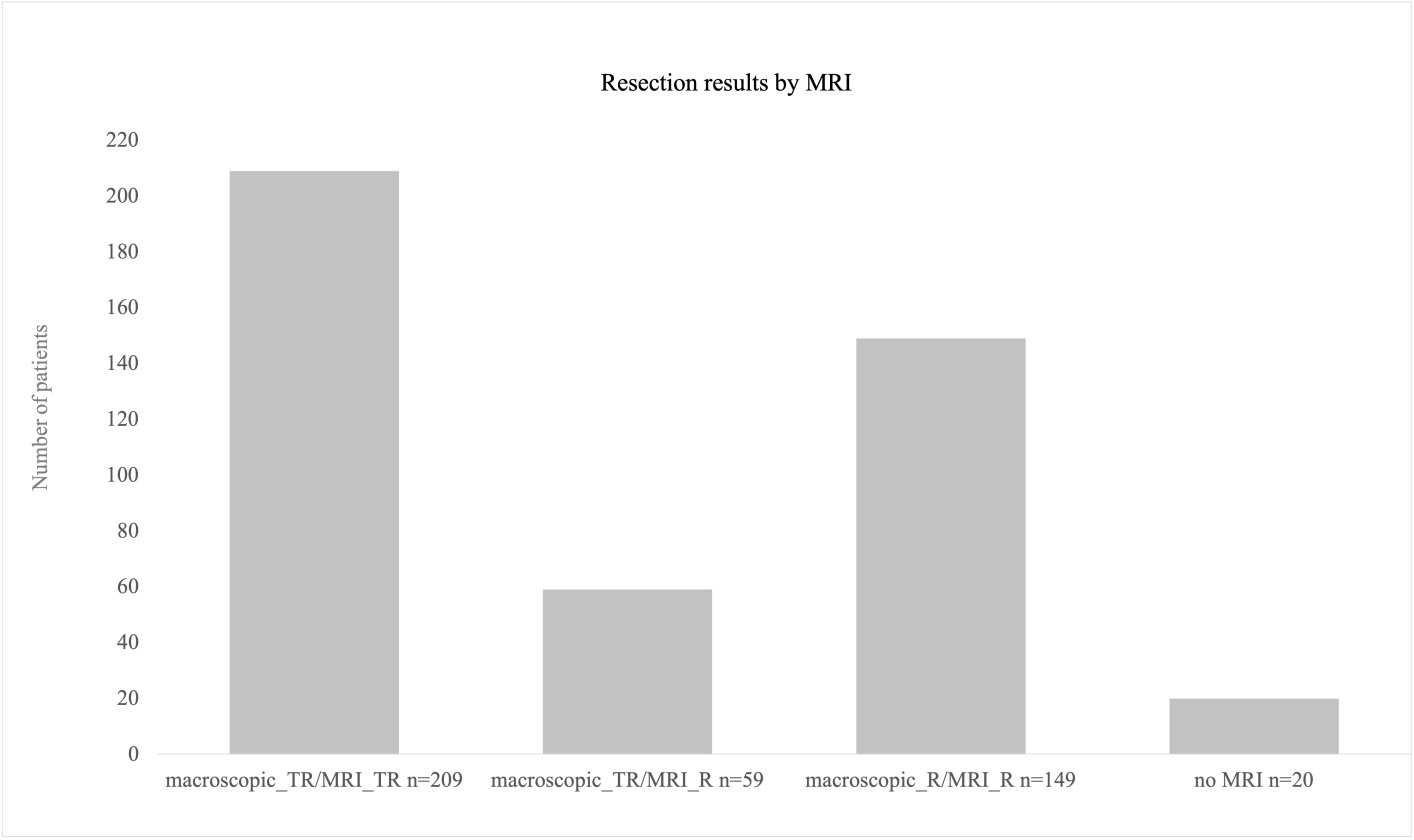
Resection results as obtained by postoperative MRI scans. A total of 209 procedures were finished with total resection. In 59 cases, surgeons’ evaluation was total resection intraoperatively but nevertheless post-OP MRI revealed residual volume (mean 0.4 ml). A total of 20 procedures were biopsies only where no MRI postoperatively was conducted.

For evaluation of resection results, 417 MRIs were available. In 20 cases, postoperative MRI was renounced due to biopsy-only procedures or postoperative bleeding with no reasonable MRI results expected. In 50% of the procedures, a total resection was achieved (n = 209); in 149 (36%) procedures, an already intraoperatively expected residual volume was proven by postoperative MRI, mostly in left fronto-temporal and straight left temporal lesions. In 14% of the cases, intraoperative evaluation of total resection failed proof by postoperative MRI. Overall, 268 surgeries were evaluated as total resection procedures by the surgeons; however, in 59 cases, postoperative MRI revealed residual tumor volume in those cases with a mean residual volume of 0.41 ml [± SD 0.73, range 0.1–4.8 ml] (**Figure 7**).

## Discussion

The present study summarizes neurological outcome and resection results of 437 procedures as well as risk factors for neurological impairment after surgical procedures in eloquent brain areas when combining all modalities of monitoring and mapping procedures for tumor resection of infiltrating lesions.

### Stimulation procedures and neurological outcome

Monopolar stimulation was conducted in the vast majority of procedures. Motor pathway mapping with cortical and subcortical monopolar stimulation was performed on lesions in both hemispheres with no significant difference. This technique is widely used for intraoperative mapping of the motor cortex (M1) and cortico-spinal tract (22, 23). Mapping of motor functions can also be carried out using bipolar stimulation (12, 24); however, it is by far not as reliable as monopolar-induced MEPs or TES-MEP.

In our clinic, bipolar stimulation for motor mapping was only performed additionally to monopolar cortical mapping in cases when monopolar mapping revealed MEP answers in more than one gyrus or if obtained MEPs showed some inconsistences. Bipolar mapping was significantly more often used on left hemispheric lesions for language mapping procedures, which is also described as standard procedure in the literature (11, 25); however, for language mapping, patients need to be awake during surgery. In our cohort, the majority of procedures were performed in the awake status and hereof the majority of lesions were located on the left hemisphere. SSEP monitoring was also used more often in left hemispheric lesions, which the authors think is a result of the majority of lesions located left hemispheric; at least 54% of the procedures were performed on lesions located in the left hemisphere. 31% of the right hemispheric lesions were also operated on in the awake status. Our group mainly addressed right hemispheric lesions under monopolar stimulation in the awake status when fine motor skills or complex motor tasks had to be monitored during surgery.

Major focus when choosing individual mapping and or monitoring modes lies in the neurological outcome. Maximum resection results shall be achieved under maximum safe circumstances concerning motor and speech integrity of the patient. New neurological deficits after brain surgery negatively affect quality of life in glioma patients, which has been discussed more frequently in the past years to be defined as an important prognostic factor (26), and can contribute to a shorter overall survival at least in patients with glioblastoma (27). Furthermore, a delay in adjuvant therapies due new postoperative motor impairment contributes to a decrease in life expectancy. Thus, there is consensus about focusing on preservation of functional integrity during surgical therapy of brain tumors especially high-grade gliomas, as they cannot be cured by surgery (28).

In our study with a large number of procedures, we only found permanent new neurological deficits in 2% of the procedures. Viagano et al. published similar results with new permanent deficits in 1.9% of patients when combining TES and DCS high-frequency stimulation in asleep procedures for tumors affecting motor pathways (29). Rossi et al. studied outcomes in 102 patients with tumors affecting the motor cortex when using different stimulation paradigms for high-frequency stimulation. In the standard approach group, using the same paradigm for monopolar stimulation as we did in our study, also 2% of the patients suffered from permanent neurological impairment (30). A meta-analysis including 90 publications with over 8,000 glioma patients revealed slightly more permanent neurological deficits with 3.4% of surgeries under mapping procedures in eloquent procedures (31). The same result with 3.4% new deficits were achieved in a study by Gogos et al. (16), comprising 58 patients with diagnosed glioma and lesions located near motor pathways.

In patients with direct postoperative neurological impairment, we found two patients in which intraoperatively an increase of SSEP latencies was seen. In three patients, speech testing showed difficulties due to lack of patients’ compliance resolving in a new deficit in the postoperative phase. In the other cases, there were no warning signs such as loss of SSEP or MEP signals, but majority of patients that underwent surgery in the awake status showed fine impairment during intraoperative testing; therefore, resection was stopped under careful consideration of the clinical findings.

None of the patients with permanent deficits underwent surgery with special intraoperative monitoring or mapping events. Five patients suffered from speech disorders, and four patients suffered from new high-grade hemiparesis. Two patients showed minor territorial infarcts in the postoperative MRI; it must be assumed that these were causal for the new and then in the follow-up also permanent neurological deficit (hemiparesis).

There are two questions to be raised in the light of the certainly very low persistent deficits in our cohort. On the one hand, the question is whether the cohorts’ localizations really were as functional as assumed from the MRI. We found that only in 0.7% of the procedures, there were only negative mapping results or there was no MEP or SSEP signal to be obtained during the surgical procedure. The authors concluded that in these cases, the tumor was not functionally located. However, this is a very small number given more than 99% of the surgeries with positive feedback and useful monitoring/mapping procedures as evaluated postoperatively by the surgeons. On the other hand, heterogeneity of our cohort might have contributed to the slightly better result as there are different growth and therefore infiltration patterns between tumor entities resulting in different complexities of functional preservation during resection. Infiltrating tumors might be relevantly of higher risk for postoperative deficits due to difficulties in resection limits. In our cohort, the infiltrating tumors were the majority, but there were a not insignificant number of patients with tumor entities that are known for not infiltrating but extruding growth patterns, which is different to the meta-analysis of de Witt et al. as they only enclosed glioma patients with infiltrating growth patterns. In order to evaluate the significance of neuropathological diagnoses, we correlated diagnoses with neurological outcome and found no significant correlation. Thus, for our cohort, we did not see a link from diagnoses to infiltration patterns, resection, and permanent deficits.

When searching for determinants that contributed to the patients’ outcome, we found that surgical procedures on left hemispheric lesions were more often noticed to cause postoperative neurological deficits than right hemispheric procedures. Nevertheless, this did not result in permanent new neurological deficits at 6 months FU. However, nine patients suffered from permanent deficit after 6 months post-op, four had recurrent surgery, and five underwent primary surgery. 67% were left hemispheric lesions with five patients suffering from speech disorder and one patient suffering from motor impairment in a left-parietal lesion. Interestingly, we found a strong correlation of preoperative neurological deficit and death within the first 6 months postsurgery.

In our study, majority of left hemispheric lesions were operated in the awake status. Awake procedures are discussed to improve safety of resection (24, 32). Although we found that there was a significant correlation between awake surgical status and direct postoperative speech deficit, which was not seen for motor impairment, in our cohort and this correlation was seen independently from the localization of the lesion, the overall 6-month evaluation of persistent deficits was not significantly influenced by the surgical status. Early postoperative overall neurological deterioration was only seen when using monopolar stimulation; all other monitoring or mapping techniques did not significantly influence the neurological outcome. However, again this finding was not verifiable at the 6-month FU and might have been influenced by the circumstance that monopolar stimulation has been used in nearly 80% of the procedures compared with only 38% bipolar stimulation; we think that the wider exposure of monopolar stimulation might have increased the probability of postoperative effect. Other statistically significant cofounders were not found.

### Intraoperative seizures

Intraoperative seizures induced by DCS are commonly seen and discussed complications in the literature. Studies provide a wide range of stimulation-induced seizures with reported rates in the low single-digit up to more than 50%, leading to an increase of neurological postoperative impairment (33–35). Part of the discussion are predictors for intraoperative seizures. Preoperative seizures tend to be risk factors for stimulation-induced intraoperative seizures (24, 33, 36). In our cohort, we recorded seizures in 8% of the procedures, with none of the patients suffering from preoperative seizures. All patients were therapy-naïve concerning anticonvulsants. We were not able to reproduce findings of correlation between preoperative and intraoperative seizures; however, in our cohort bipolar stimulation expectedly correlated with incidence of stimulation-induced seizures, whereas stimulation intensity did not significantly influence the incidence of intraoperative seizures.

### Evaluation of surgeons

One of the major aims of this study was to correlate preoperatively chosen monitoring or mapping techniques with postoperative evaluation of the techniques used by the surgeon. We found that in 73% procedures, the postoperative evaluation of obligatory stimulation mode matched the preoperatively defined methods to be used intraoperatively. The localization of the tumor correlated with postoperative surgeons’ evaluation, and as expected, monopolar obligatory mapping was found to be essential more often in right hemispheric lesions, whereas bipolar mapping and combined monopolar/bipolar mapping were more often evaluated as essential for tumors in the left hemisphere. Interestingly, SSEP/MEP monitoring only or in combination with DCS was only seen obligatory in 15% with a rising number of obligatory evaluations after engagement of new monitoring staff from 11 procedures that were evaluated as obligatory monitored by SSEP/MEP to 25 procedures (per year). The authors discussed that and found that there either must be a bias due to availability of more monitoring staff or procedures became more demanding with high-risk vascular involvement. Nevertheless, there was no correlation between stated obligatory stimulation mode and postoperative neurological outcome. Interestingly, more incongruence between the evaluated obligatory method and actual technique used was seen with bipolar mapping. This might be a result of non-adequate awake patients with left hemispheric lesions that needed to undergo bipolar mapping due to localization, but mapping or adequate testing was not able to be performed due to noncompliance to awake situation or seizures at the beginning.

### Resection result

The extent of resection and its impact on overall survival (OS) in patients suffering from glioma are widely discussed. Different thresholds for impact on OS were published ranging from 60% to 98% (37–40). Also, in oligodendroglioma and metastasis, the extent of resection seems to have a significant impact on survival (41, 42).

Total resection, meaning no detectable contrast enhancement in the post-OP MRI, was achieved in 50% of the procedures. There were 20 procedures performed as biopsy without post-OP MRI. In 149 (36%) procedures, the functional limit was achieved intraoperatively, as defined by monitoring and or mapping results. In 59 (14%) procedures, surgeons assumed total resection but post-OP MRI showed residual tumor volume with a very low mean residual volume of 0.41 ml and a maximum residual volume of 4.8 ml in one case. In our cohort, total resection was achieved in arguably fewer cases than in comparable publications (31), but there are some points that led to this result. Firstly, functional limits were achieved intraoperatively, in more than one-third of procedures. Achievement of total resection would have meant neurological deterioration for the patient, something that has to be avoided in the light of survival benefits. Secondly, using intraoperative tools for functional preservation and then deciding intraoperatively to maximize resection regardless of the mapping results would fail the surgical aim. Thirdly, in the procedures that were evaluated as total resection but nonetheless showed residual volume in the post-OP MRI, residual volume was marginal with a mean of 0.4 ml. Concerning comparable publications, a residual tumor volume up to 8 cm^3^ could be acceptable for an effect on survival that can still be achieved at least for gliomas (40). Furthermore, with this study, we searched for impacts on resection results but found that there was no significant correlation either between monitoring/mapping results or between the resection result and neurological outcome. However, we did not analyze survival data of the present cohort. Nevertheless, in consideration of already published literature, we assume that the very much marginal residual volume did not have any negative impact on patients’ OS.

### Limitation

The lacking survival data might be a limitation to the study in order to comprehend the given resection results. Nonetheless, as this was not the focus of the present analysis, the authors renounced this fractional analysis. Additionally in some cases, information on stimulation modes or thresholds could not be obtained from all sources that were available to the authors. However, as there were only minor missing data, we do not think that this would have affected the results significantly.

In the context of determining and evaluating the extent of resection in patients with glioblastoma, another limitation might be the lack of an assessment of the influence of 5-ALA on the resection. However, a meaningful statistical analysis in the reported cohort was not feasible because all patients with suspected or confirmed brain tumors, and at least at the beginning of the observation period, patients suspected of cerebral metastasis, received 5-ALA. Therefore, group comparisons regarding the extent of resection for this cohort were not applicable. We clearly assume that, as reported in the literature, resection under 5-ALA had a positive impact on conduction of resection. However, it is important to consider that the current cohort consists of highly functionally located tumors. Even though resection was performed under fluorescence guidance, and residual fluorescence may have been visible, functional assessment was more decisive for the extent of resection.

## Conclusion

In the light of the important role of surgical procedures in the therapy process for brain tumor lesions and the superior aim to preserve functionality of the patients, adequate planning of intraoperative required monitoring or mapping techniques is of highest priority. Deciding which intraoperative mapping and or monitoring procedure is best for the patient is highly individual. The choice of a certain technique mainly depends on localization and experience of the surgeon. With the present study, we demonstrate operability of highly functional infiltrating brain lesions of various localizations without major neurological impairment under usage of IONM and mapping techniques. We were able to give an overview of pitfalls and strengths of the different technical procedures and if, respectively, how they correlate with postoperative neurological outcome and resection results. Furthermore, we retrospectively included the surgeons’ view and evaluated the impact of a possibly existing mismatch between preoperative and postoperative assessment of individual technical considerations for each procedure. With this evaluation, we were able to show that certain techniques might not be useful for every case and in the light of optimalization of resources not required for safe resections in every cases. These results shall contribute to a practical but high-quality decision-making process for every surgeon addressing eloquent brain lesions.

## Data availability statement

The raw data supporting the conclusions of this article will be made available by the authors, without undue reservation.

## Ethics statement

The studies involving humans were approved by Ethikkommission Düsseldorf, Heinrich-Heine Universität. The studies were conducted in accordance with the local legislation and institutional requirements. The ethics committee/institutional review board waived the requirement of written informed consent for participation from the participants or the participants’ legal guardians/next of kin because Local laws permit usage of clinical data that was obtained during routine procedures without informed consent.

## Author contributions

FS-B contributed to the design and implementation of the research, performed data collection and designed statistical analysis of the results and wrote the manuscript. MR contributed to the design of the research and data collection and to the writing of the manuscript. MS designed and directed the project and contributed to the writing of the manuscript. All authors contributed to the article and approved the submitted version.

## References

[1] Stummer W Reulen HJ Meinel T Pichlmeier U Schumacher W Tonn JC. Extent of resection and survival in glioblastoma multiforme: identification of and adjustment for bias. Neurosurgery (2008) 62(3):564–76. doi: 10.1227/01.neu.0000317304.31579.17 18425006

[2] Sanai N Polley MY McDermott MW Parsa AT Berger MS. An extent of resection threshold for newly diagnosed glioblastomas. J Neurosurg (2011) 115(1):3–8. doi: 10.3171/2011.2.JNS10998 21417701

[3] Brown TJ Brennan MC Li M Church EW Brandmeir NJ Rakszawski KL. Association of the extent of resection with survival in glioblastoma: A systematic review and meta-analysis. JAMA Oncol (2016) 2(11):1460–9. doi: 10.1001/jamaoncol.2016.1373 PMC6438173 27310651

[4] Berger MS Deliganis AV Dobbins J Keles GE. The effect of extent of resection on recurrence in patients with low grade cerebral hemisphere gliomas. Cancer (1994) 74(6):1784–91. doi: 10.1002/1097-0142(19940915)74:6<1784::AID-CNCR2820740622>3.0.CO;2-D 8082081

[5] Smith JS Chang EF Lamborn KR Chang SM Prados MD Cha S. Role of extent of resection in the long-term outcome of low-grade hemispheric gliomas. J Clin Oncol (2008) 26(8):1338–45. doi: 10.1200/JCO.2007.13.9337 18323558

[6] Prabhu RS Press RH Patel KR Boselli DM Symanowski JT Lankford SP. Single-fraction stereotactic radiosurgery (SRS) alone versus surgical resection and SRS for large brain metastases: A multi-institutional analysis. Int J Radiat Oncol Biol Phys (2017) 99(2):459–67. doi: 10.1016/j.ijrobp.2017.04.006 28871997

[7] Patchell RA Tibbs PA Walsh JW Dempsey RJ Maruyama Y Kryscio RJ. A randomized trial of surgery in the treatment of single metastases to the brain. N Engl J Med (1990) 322(8):494–500. doi: 10.1056/NEJM199002223220802 2405271

[8] Ferracci FX Duffau H. Improving surgical outcome for gliomas with intraoperative mapping. Expert Rev Neurother (2018) 18(4):333–41. doi: 10.1080/14737175.2018.1451329 29521555

[9] Keles GE Lundin DA Lamborn KR Chang EF Ojemann G Berger MS. Intraoperative subcortical stimulation mapping for hemispherical perirolandic gliomas located within or adjacent to the descending motor pathways: evaluation of morbidity and assessment of functional outcome in 294 patients. J Neurosurg (2004) 100(3):369–75. doi: 10.3171/jns.2004.100.3.0369 15035270

[10] Kombos T Suess O Ciklatekerlio O Brock M. Monitoring of intraoperative motor evoked potentials to increase the safety of surgery in and around the motor cortex. J Neurosurg (2001) 95(4):608–14. doi: 10.3171/jns.2001.95.4.0608 11596955

[11] Bello L Gallucci M Fava M Carrabba G Giussani C Acerbi F. Intraoperative subcortical language tract mapping guides surgical removal of gliomas involving speech areas. Neurosurgery (2007) 60(1):67–80. discussion 80-62. doi: 10.1227/01.NEU.0000249206.58601.DE 17228254

[12] Duffau H Capelle L Denvil D Sichez N Gatignol P Taillandier L. Usefulness of intraoperative electrical subcortical mapping during surgery for low-grade gliomas located within eloquent brain regions: functional results in a consecutive series of 103 patients. J Neurosurg (2003) 98(4):764–78. doi: 10.3171/jns.2003.98.4.0764 12691401

[13] Tomasino B Guarracino I Ius T Skrap M. Continuous real-time neuropsychological testing during resection phase in left and right prefrontal brain tumors. Curr Oncol (2023) 30(2):2007–20. doi: 10.3390/curroncol30020156 PMC9955514 36826117

[14] Duffau H. Awake surgery for nonlanguage mapping. Neurosurgery (2010) 66(3):523–8. discussion 528-529. doi: 10.1227/01.NEU.0000364996.97762.73 20173547

[15] Ruis C. Monitoring cognition during awake brain surgery in adults: A systematic review. J Clin Exp Neuropsychol (2018) 40(10):1081–104. doi: 10.1080/13803395.2018.1469602 30067443

[16] Gogos AJ Young JS Morshed RA Avalos LN Noss RS Villanueva-Meyer JE. Triple motor mapping: transcranial, bipolar, and monopolar mapping for supratentorial glioma resection adjacent to motor pathways. J Neurosurg (2020) 134(6):1728–37. doi: 10.3171/2020.3.JNS193434 32502996

[17] Seidel K Szelényi A Bello L. Chapter 8 - Intraoperative mapping and monitoring during brain tumor surgeries. In: Nuwer MR MacDonald DB, editors. Handbook of Clinical Neurology. Amsterdam: Elsevier (2022). p. 133–49. 10.1016/B978-0-12-819826-1.00013-2 35772883

[18] Schucht P Seidel K Jilch A Beck J Raabe A. A review of monopolar motor mapping and a comprehensive guide to continuous dynamic motor mapping for resection of motor eloquent brain tumors. Neurochirurgie (2017) 63(3):175–80. doi: 10.1016/j.neuchi.2017.01.007 28506487

[19] Verst SM de Aguiar PHP Joaquim MAS Vieira VG Sucena ABC Maldaun MVC. Monopolar 250-500Hz language mapping: Results of 41 patients. Clin Neurophysiol Pract (2019) 4:1–8. doi: 10.1016/j.cnp.2018.11.002 30619979 PMC6312792

[20] Louis DN Perry A Wesseling P Brat DJ Cree IA Figarella-Branger D. The 2021 WHO classification of tumors of the central nervous system: a summary. Neuro Oncol (2021) 23(8):1231–51. doi: 10.1093/neuonc/noab106 PMC8328013 34185076

[21] Huber W Poeck K Willmes K. The aachen aphasia test. Adv Neurol (1984) 42:291–303. 6209953

[22] Schucht P Seidel K Beck J Murek M Jilch A Wiest R. Intraoperative monopolar mapping during 5-ALA-guided resections of glioblastomas adjacent to motor eloquent areas: evaluation of resection rates and neurological outcome. Neurosurg Focus (2014) 37(6):E16. doi: 10.3171/2014.10.FOCUS14524 25434385

[23] Bello L Riva M Fava E Ferpozzi V Castellano A Raneri F. Tailoring neurophysiological strategies with clinical context enhances resection and safety and expands indications in gliomas involving motor pathways. Neuro Oncol (2014) 16(8):1110–28. doi: 10.1093/neuonc/not327 PMC4096171 24500420

[24] Hervey-Jumper SL Li J Lau D Molinaro AM Perry DW Meng L. Awake craniotomy to maximize glioma resection: methods and technical nuances over a 27-year period. J Neurosurg (2015) 123(2):325–39. doi: 10.3171/2014.10.JNS141520 25909573

[25] Ojemann G Ojemann J Lettich E Berger M. Cortical language localization in left, dominant hemisphere. An electrical stimulation mapping investigation in 117 patients. 1989. J Neurosurg (2008) 108(2):411–21. doi: 10.3171/JNS/2008/108/2/0411 18240946

[26] Cheng JX Zhang X Liu BL. Health-related quality of life in patients with high-grade glioma. Neuro Oncol (2009) 11(1):41–50. doi: 10.1215/15228517-2008-050 18628405 PMC2718958

[27] McGirt MJ Mukherjee D Chaichana KL Than KD Weingart JD Quinones-Hinojosa A. ASSOCIATION OF SURGICALLY ACQUIRED MOTOR AND LANGUAGE DEFICITS ON OVERALL SURVIVAL AFTER RESECTION OF GLIOBLASTOMA MULTIFORME. Neurosurgery (2009) 65(3):463–70. doi: 10.1227/01.NEU.0000349763.42238.E9 19687690

[28] Weller M van den Bent M Preusser M Le Rhun E Tonn JC Minniti G. EANO guidelines on the diagnosis and treatment of diffuse gliomas of adulthood. Nat Rev Clin Oncol (2021) 18(3):170–86. doi: 10.1038/s41571-020-00447-z PMC7904519 33293629

[29] Viganò L Callipo V Lamperti M Rossi M Conti Nibali M Sciortino T. Transcranial versus direct electrical stimulation for intraoperative motor-evoked potential monitoring: Prognostic value comparison in asleep brain tumor surgery. Front Oncol (2022) 12:963669. doi: 10.3389/fonc.2022.963669 36249008 PMC9557724

[30] Rossi M Nibali MC Viganò L Puglisi G Howells H Gay L. Resection of tumors within the primary motor cortex using high-frequency stimulation: oncological and functional efficiency of this versatile approach based on clinical conditions. J Neurosurg (2019) p:1. doi: 10.3171/2019.5.JNS19453 31398706

[31] De Witt Hamer PC Robles SG Zwinderman AH Duffau H Berger MS. Impact of intraoperative stimulation brain mapping on glioma surgery outcome: a meta-analysis. J Clin Oncol (2012) 30(20):2559–65. doi: 10.1200/JCO.2011.38.4818 22529254

[32] Taylor MD Bernstein M. Awake craniotomy with brain mapping as the routine surgical approach to treating patients with supratentorial intraaxial tumors: a prospective trial of 200 cases. J Neurosurg (1999) 90(1):35–41. doi: 10.3171/jns.1999.90.1.0035 10413153

[33] Roca E Pallud J Guerrini F Panciani PP Fontanella M Spena G. Stimulation-related intraoperative seizures during awake surgery: a review of available evidences. Neurosurg Rev (2020) 43(1):87–93. doi: 10.1007/s10143-019-01214-0 31797239

[34] Spena G Schucht P Seidel K Rutten GJ Freyschlag CF D’Agata F. Brain tumors in eloquent areas: A European multicenter survey of intraoperative mapping techniques, intraoperative seizures occurrence, and antiepileptic drug prophylaxis. Neurosurg Rev (2017) 40(2):287–98. doi: 10.1007/s10143-016-0771-2 27481498

[35] Ulkatan S Jaramillo AM Tellez MJ Kim J Deletis V Seidel K. Incidence of intraoperative seizures during motor evoked potential monitoring in a large cohort of patients undergoing different surgical procedures. J Neurosurg (2017) 126(4):1296–302. doi: 10.3171/2016.4.JNS151264 27341047

[36] Boetto J Bertram L Moulinié G Herbet G Moritz-Gasser S Duffau H. Low rate of intraoperative seizures during awake craniotomy in a prospective cohort with 374 supratentorial brain lesions: electrocorticography is not mandatory. World Neurosurg (2015) 84(6):1838–44. doi: 10.1016/j.wneu.2015.07.075 26283485

[37] Grabowski MM Recinos PF Nowacki AS Schroeder JL Angelov L Barnett GH. Residual tumor volume versus extent of resection: predictors of survival after surgery for glioblastoma. J Neurosurg (2014) 121(5):1115–23. doi: 10.3171/2014.7.JNS132449 25192475

[38] Chaichana KL Jusue-Torres I Navarro-Ramirez R Raza SM Pascual-Gallego M Ibrahim A. Establishing percent resection and residual volume thresholds affecting survival and recurrence for patients with newly diagnosed intracranial glioblastoma. Neuro Oncol (2014) 16(1):113–22. doi: 10.1093/neuonc/not137 PMC3870832 24285550

[39] Lacroix M Abi-Said D Fourney DR Gokaslan ZL Shi W DeMonte F. A multivariate analysis of 416 patients with glioblastoma multiforme: prognosis, extent of resection, and survival. J Neurosurg (2001) 95(2):190–8. doi: 10.3171/jns.2001.95.2.0190 11780887

[40] Coburger J Segovia J Ganslandt O Ringel F Wirtz CR Renovanz M. Counseling patients with a glioblastoma amenable only for subtotal resection: results of a multicenter retrospective assessment of survival and neurologic outcome. World Neurosurg (2018) 114:e1180–5. doi: 10.1016/j.wneu.2018.03.173 29621607

[41] Kinslow CJ Garton ALA Rae AI Marcus LP Adams CM McKhann GM. Extent of resection and survival for oligodendroglioma: a U.S. population-based study. J Neurooncol (2019) 144(3):591–601. doi: 10.1007/s11060-019-03261-5 31407129

[42] Winther RR Hjermstad MJ Skovlund E Aass N Helseth E Kaasa S. Surgery for brain metastases-impact of the extent of resection. Acta Neurochir (Wien) (2022) 164(10):2773–80. doi: 10.1007/s00701-021-05104-7 PMC9519668 35080651

