# Supplementary material for: Feasibility of intraoperative neuromonitoring and cortical/subcortical mapping in patients with cerebral lesions of highly functional localizations—pathway to case adapted monitoring and mapping procedures
Source: Front Oncol. 2023 Nov 23;13:1235212. doi: 10.3389/fonc.2023.1235212 (PMC10702247; doi:10.3389/fonc.2023.1235212)
Supplement: Supplementary file 1 [file DataSheet_1.pdf]

Supplements to the manuscript

Feasibility of intraoperative neuromonitoring and cortical/subcortical mapping in patients with cerebral lesions of highly functional localizations- pathway to case adapted monitoring and mapping procedures

Franziska Staub-Bartelt, Marion Rapp, Michael Sabel
Department of Neurosurgery, University Hospital Duesseldorf, Germany

MORE DETAILED DESCRIPTION OF INTRAOPERATIVE MONITORING AND MAPPING TECHNIQUES

There are different methods used for intraoperative monitoring (IOM) of neurological function. Transcranial electric stimulation (TES) can be used for motor evoked potentials (TES-MEP). MEPs can be used for monitoring of motor pathway integrity. Different warning signs are discussed in the literature with decrease of amplitude of more than 50% being widely used but also threshold increases for triggering MEPs are considered as warning sign during monitoring motor function [43]. Additionally somatosensory evoked potential (SSEP) monitoring can be performed. SSEP monitor function of sensory pathways by measuring latency and amplitude of signals between two determined points: stimulation of peripheral nerves and recording of the transmitted signal peripheral or centrally from the cortex. Detoriation of SSEP defined as prolongation of latency and decrease of amplitude are considered as warning signs [44].

MEP monitoring can also be performed via direct cortical stimulation (DCS) by placing a strip electrode (SE) on the precentral gyrus allowing a continuous control of neurological motor function in asleep patients. DCS is also carried out by the usage of handheld monopolar or bipolar stimulation probes [16, 17], which enables the surgeon to map the functionality of the tissue.

Bipolar stimulation has been introduced by Walter Penfield in the late 30ies of the 20th century [45] and has been referred to a low frequency stimulation with long stimulation duration. To date it is commonly used for awake mapping procedures but can in some cases also be very useful in motor mapping [23] in the asleep setting. Even though bipolar mapping combined with Electromyographie for MEP monitoring is far from reliable compared to monopolar induced MEPs. Therefore, monopolar mapping is particularly used for motor mapping for tumors located near or around motor pathways even though speech mapping is also performed at least on a research basis via monopolar mapping stimulation [18, 19].

Besides an anodal-cathodal cortical stimulation in order to define motor-thresholds, subcortical stimulation can also be performed either with a monopolar or bipolar stimulation probe. Subcortical mapping in high-frequency technique using a monopolar probe helps with estimation of distance to motor-eloquent structures during resection. Bipolar subcortical stimulation is performed for speech testing as described.

TECHNICAL MONITORING AND MAPPING DATA FOR THE COHORT

Monitoring and mapping data were obtained using the following technical devices with described standard set up for different monitoring/mapping techniques.

ISIS Xpert & C2 Xplore (inomed Medizintechnik GmbH, Emmending, Germany, Neuro Explorer Software Version 6)

Monitoring

SSEP (ISIS only): N.tibialis and N.medianus

stimulation: 0.1-20 mA, 0.5 ms pulsewidth, 1 pulse, 4.7 Hz

recording: bandpassfilter 3-500 Hz

TES-MEP (ISIS only)

stimulation: 10-220 mA, 0.5 ms pulsewidth, train of 5 with interstimulus interval of 3ms, 0.5 Hz

Recording: bandpassfilter 95-2000 Hz extremities, 195-2000 face

DCS MEP (ISIS only, 4-6 contact subdural strip)

stimulation: 0.1- 30 mA, 0.5 ms pulsewidth, train of 5 with interstimulus interval of 3ms, 0.5 Hz

recording: bandpassfilter 95-2000 Hz extremities, 195-2000 face

Mapping

Cortical and subcortical with monopolar probe (high-frequency)

stimulation: 0.1-30 mA, 0.5 ms pulsewidth, train of 5 with interstimulus interval of 3ms, 0.5 Hz

recording: bandpassfilter 95-2000 Hz extremities, 195-2000 face

cortical and subcortical with bipolar probe (low-frequency)

stimulation: 0.5-4 mA, 0.8 ms pulsewidth, 1 pulse, 60 Hz, **4 s stimulation**

recordings: only in the context of triggered EMG recordings

In cases where the C2 Xplore device was used, amperage of bipolar stimulation is given in the numbers of ISIS Xpert device as there is are other technical nuances between those devices leading to different settings. For better comparison we standardized the data obtained.

Ojeman Cortical Stimulator (Integra LifeSciences,

Mapping

Cortical and subcortical with bipolar probe (2 balltips with 5 mm distance), 0.1-4 mA, 60 Hz

Figures

Figure_Suppl_1

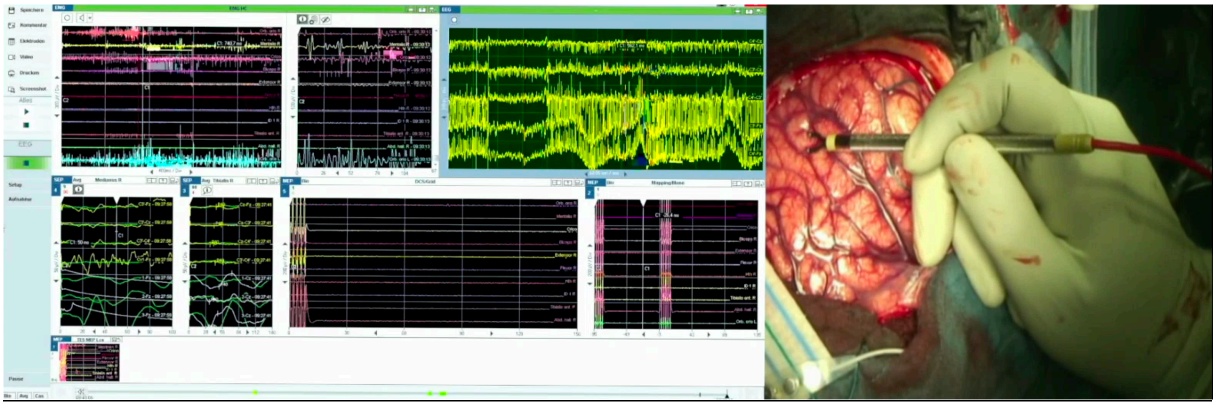

Figure_suppl_1: Intraoperative example of bipolar mapping techniques. A bipolar stimulation probe is placed on the cortex. At the same time, the patient has to fulfill different language tasks. The monitor shows strong muscle potential due to the awake and speaking patient.

Figure_Suppl_2

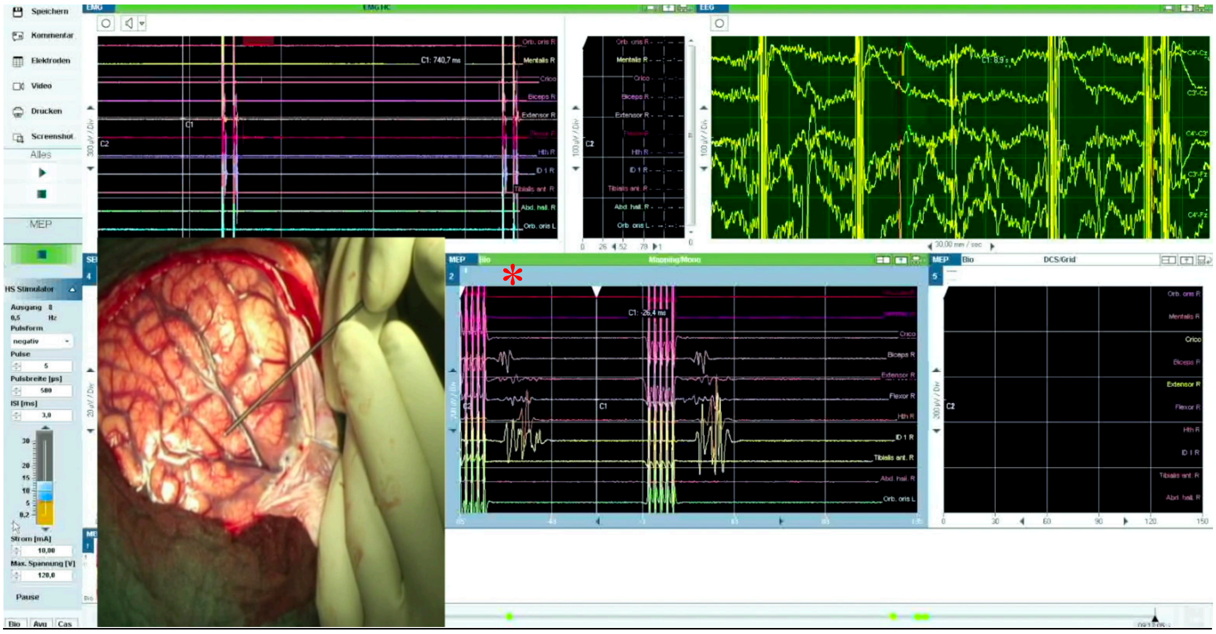

Figure_suppl_1: Intraoperative example of monopolar mapping techniques. The probe is placed on the cortex. The technician choses amplitude of stimulation at the device. In this patient we obtained cortical and subcortical MEPs with 10 mA (red * = MEP BOX, MEPS for upper and lower extremities).

STROBE Statement—checklist of items that should be included in reports of observational studies

	Item No.	Recommendation	Page No.
**Title and abstract**	1	(*a*) Indicate the study's design with a commonly used term in the title or the abstract	1
		(*b*) Provide in the abstract an informative and balanced summary of what was done and what was found	2
**Introduction**			
Background/rationale	2	Explain the scientific background and rationale for the investigation being reported	3-5
Objectives	3	State specific objectives, including any prespecified hypotheses	4-5
**Methods**			
Study design	4	Present key elements of study design early in the paper	6-9
Setting	5	Describe the setting, locations, and relevant dates, including periods of recruitment, exposure, follow-up, and data collection	6
Participants	6	(*a*) *Cohort study*—Give the eligibility criteria, and the sources and methods of selection of participants. Describe methods of follow-up<br>*Case-control study*—Give the eligibility criteria, and the sources and methods of case ascertainment and control selection. Give the rationale for the choice of cases and controls<br>*Cross-sectional study*—Give the eligibility criteria, and the sources and methods of selection of participants	6
		(*b*) *Cohort study*—For matched studies, give matching criteria and number of exposed and unexposed<br>*Case-control study*—For matched studies, give matching criteria and the number of controls per case	n.a.<br>n.a.
Variables	7	Clearly define all outcomes, exposures, predictors, potential confounders, and effect modifiers. Give diagnostic criteria, if applicable	7-8
Data sources/ measurement	8*	For each variable of interest, give sources of data and details of methods of assessment (measurement). Describe comparability of assessment methods if there is more than one group	
Bias	9	Describe any efforts to address potential sources of bias	6-8
Study size	10	Explain how the study size was arrived at	6

Quantitative variables	11	Explain how quantitative variables were handled in the analyses. If applicable, describe which groupings were chosen and why	
Statistical methods	12	(*a*) Describe all statistical methods, including those used to control for confounding	9
		(*b*) Describe any methods used to examine subgroups and interactions	9
		(*c*) Explain how missing data were addressed	8
		(*d*) *Cohort study*—If applicable, explain how loss to follow-up was addressed<br>*Case-control study*—If applicable, explain how matching of cases and controls was addressed<br>*Cross-sectional study*—If applicable, describe analytical methods taking account of sampling strategy	
		(*e*) Describe any sensitivity analyses	
**Results**			
Participants	13*	(a) Report numbers of individuals at each stage of study—eg numbers potentially eligible, examined for eligibility, confirmed eligible, included in the study, completing follow-up, and analysed	10-13
		(b) Give reasons for non-participation at each stage	n.a.
		(c) Consider use of a flow diagram	
Descriptive data	14*	(a) Give characteristics of study participants (eg demographic, clinical, social) and information on exposures and potential confounders	Table 1
		(b) Indicate number of participants with missing data for each variable of interest	10-13+figures
		(c) *Cohort study*—Summarise follow-up time (eg, average and total amount)	
Outcome data	15*	*Cohort study*—Report numbers of outcome events or summary measures over time	*10.13*
		Case-control study—Report numbers in each exposure category, or summary measures of exposure	
		Cross-sectional study—Report numbers of outcome events or summary measures	
Main results	16	(*a*) Give unadjusted estimates and, if applicable, confounder-adjusted estimates and their precision (eg, 95% confidence interval). Make clear which confounders were adjusted for and why they were included	10.13
		(*b*) Report category boundaries when continuous variables were categorized	
		(*c*) If relevant, consider translating estimates of relative risk into absolute risk for a meaningful time period	

Other analyses	17	Report other analyses done—eg analyses of subgroups and interactions, and sensitivity analyses	
**Discussion**			
Key results	18	Summarise key results with reference to study objectives	14
Limitations	19	Discuss limitations of the study, taking into account sources of potential bias or imprecision. Discuss both direction and magnitude of any potential bias	17
Interpretation	20	Give a cautious overall interpretation of results considering objectives, limitations, multiplicity of analyses, results from similar studies, and other relevant evidence	14-17

Generalisability	21	Discuss the generalisability (external validity) of the study results	17
**Other information**			
Funding	22	Give the source of funding and the role of the funders for the present study and, if applicable, for the original study on which the present article is based	n.a.

*Give information separately for cases and controls in case-control studies and, if applicable, for exposed and unexposed groups in cohort and cross-sectional studies.

Note: An Explanation and Elaboration article discusses each checklist item and gives methodological background and published examples of transparent reporting. The STROBE checklist is best used in conjunction with this article (freely available on the Web sites of PLoS Medicine at http://www.plosmedicine.org/, Annals of Internal Medicine at http://www.annals.org/, and Epidemiology at http://www.epidem.com/). Information on the STROBE Initiative is available at www.strobe-statement.org.
